# Templated Assembly of Collagen Fibers Directs Cell Growth in 2D and 3D

**DOI:** 10.1038/s41598-017-10182-8

**Published:** 2017-08-29

**Authors:** G. Y. Liu, R. Agarwal, K. R Ko, M. Ruthven, H. T. Sarhan, J. P. Frampton

**Affiliations:** School of Biomedical Engineering, Dalhousie University, Halifax, Nova Scotia Canada

## Abstract

Collagen is widely used in tissue engineering and regenerative medicine, with many examples of collagen-based biomaterials emerging in recent years. While there are numerous methods available for forming collagen scaffolds from isolated collagen, existing biomaterial processing techniques are unable to efficiently align collagen at the microstructural level, which is important for providing appropriate cell recognition and mechanical properties. Although some attention has shifted to development of fiber-based collagen biomaterials, existing techniques for producing and aligning collagen fibers are not appropriate for large-scale fiber manufacturing. Here, we report a novel biomaterial fabrication approach capable of efficiently generating collagen fibers of appropriate sizes using a viscous solution of dextran as a dissolvable template. We demonstrate that myoblasts readily attach and align along 2D collagen fiber networks created by this process. Furthermore, encapsulation of collagen fibers with myoblasts into non-cell-adherent hydrogels promotes aligned growth of cells and supports their differentiation. The ease-of-production and versatility of this technique will support future development of advanced *in vitro* tissue models and materials for regenerative medicine.

## Introduction

The organization of collagen in the extracellular matrix imparts specific tensile properties to tissues and serves as a determinant of tissue architecture^1^. There are 29 known collagen sub-types that contribute to tissue structure and function, of which Types I, II, III, IV, and IX are the five most abundant sub-types found in the human body^1–3^. All collagen sub-types assemble into triple helical structures formed from three left-handed α-helices that twist around each other into a right-handed tropo-collagen helix^4^. Each α-helix consists of Gly-X-Y amino acid repeats that promote collagen fibril self-assembly and provide sites for inter-molecular and interfibrillar cross-linking^5–7^. Tightly packed 30–100 nm-thick collagen fibrils assemble into collagen fibers that are 1–20 µm thick and of indeterminate length^8–10^. Appropriate organization of Type I collagen fibers is essential for maintaining the mechanical integrity and normal function of a variety of tissues including tendons, ligaments, bone, skin, and muscle^11–13^.

The pivotal role that collagen plays in determining tissue structure and function has motivated its widespread use as a biomaterial for tissue engineering and regenerative medicine. Collagen is relatively easy to isolate from a variety of tissue sources (e.g., bovine, porcine, rodent and fish collagens are commercially available) and can be produced as a recombinant human protein^14–16^. When appropriately processed, it displays low immunogenicity and retains its ability to assemble into stable fibers^17, 18^. Isolated collagen promotes integrin-dependent cell adhesion^19, 20^, and in contrast to most synthetic biomaterials, it can be remodelled by cells^21^. Isolated collagen preparations can also be readily processed into hydrogels and freeze-dried porous sponges by various top-down, or scaffold-based, biomaterial manufacturing processes^22, 23^.

Although collagen hydrogels and sponges support cell engraftment, it can be difficult to control collagen fiber alignment and cell organization in these materials. Attempts have been made to promote cell alignment by physically shaping the external macrostructure of collagen hydrogels using polydimethylsiloxane molds^24, 25^. However, collagen casting techniques are incapable of modifying the microstructural alignment of collagen fibers within hydrogels. Various forms of physical manipulation including exposure to strain^26, 27^, fluid shear^28–30^, and electromagnetic fields^29, 31, 32^ have been explored to improve collagen fiber alignment within hydrogels, but these approaches are all potentially limited by the amount of material that can be processed at a given time and the requirement of relatively sophisticated instrumentation for controlling the fiber alignment, e.g., mechanical setups, flow cells, and powerful magnetic systems. Attempts have also been made to manipulate the microstructural alignment of collagen sponges by modifying the freeze-drying process to produce discontinuous ellipsoid pores, but the effect on collagen fiber alignment is minimal and trace amounts of porogens may remain in the final collagen biomaterial^33–35^.

Due to the limitations of these top-down biomaterial manufacturing processes, there has been increased interest in bottom-up, or fiber-based, collagen biomaterial manufacturing. Electrospinning of collagen has attracted considerable attention in recent years, because this process is capable of producing nanofiber structures that resemble native collagen fibrils, and offers the ability to manipulate the porosity, structure, and orientation of the collagen fiber network^36, 37^. While ordered fiber networks and sufficient porosity for cellular infiltration can be difficult to achieve due to the random nature of electrospun fiber deposition, methods have been developed to increase alignment and pore size (e.g., the addition of sacrificial fibers)^38, 39^. However, these processes require highly specialized electrospinning equipment. In addition, harsh solvents, such as fluoroalcohols, are often used to stabilize jets of dissolved collagen during the electrospinning process^39^. Unique characteristics of these solvents allow them to optimally evaporate from self-assembling collagen fibers and minimize wet fiber deposition. However, solvent exposure can denature collagen and increase its solubility upon subsequent contact with aqueous environments^40–42^. The electrospinning process also exposes collagen to high shear forces, which can limit its ability to self-assemble into a stable conformation^42^. Electrospun collagen fibers must therefore be crosslinked prior to use as a biomaterial to avoid collagen dissolution in aqueous environments, which is not ideal because residual crosslinking agents may interfere with downstream applications. Extrusion-based collagen fibrogenesis can also produce self-assembled collagen fibers and does not require electrostatic forces or harsh solvents^17^. However, this process is slow and only produces one relatively thick fiber at a time. Attempts have been made to streamline this technique and generate aligned collagen networks, but once again the use of specialized equipment and the thickness of the fibers that are formed limit this method^43^. Finally, there have been several examples of collagen fiber assembly in microfluidic devices. While effective at promoting organization and alignment of collagen fibers of appropriate sizes, these devices are limited in terms of throughput and the ability to retrieve the fibers for downstream applications^44, 45^.

Previously, we reported a novel biomaterial fabrication approach that utilizes highly viscous dextran solutions to form thin fibers when stretched between two substrates^46^. These fibers can be layered on a collector to form organized fabrics with controlled fiber alignment, overcoming many of the limitations of other fiber-based approaches. Depending on the number of fiber layers and orientation within the dextran fabrics, unique mechanical properties can be achieved. We previously demonstrated that fabrics formed from dextran fibers are also capable of releasing bioactive agents as the fabrics dissolve. Here, we apply this biomaterial fabrication approach to develop a simple, rapid, and versatile method for generating collagen fibers of appropriate sizes that can be organized into networks to support the two-dimensional (2D) and three-dimensional (3D) growth of skeletal muscle cells. Our manufacturing process produces an accessible and tunable collagen-based material that may hold promise as a matrix for supporting the growth of a wide-variety of additional cell types, and as a material for wound healing and reconstruction.

## Results

**Figure 1:**
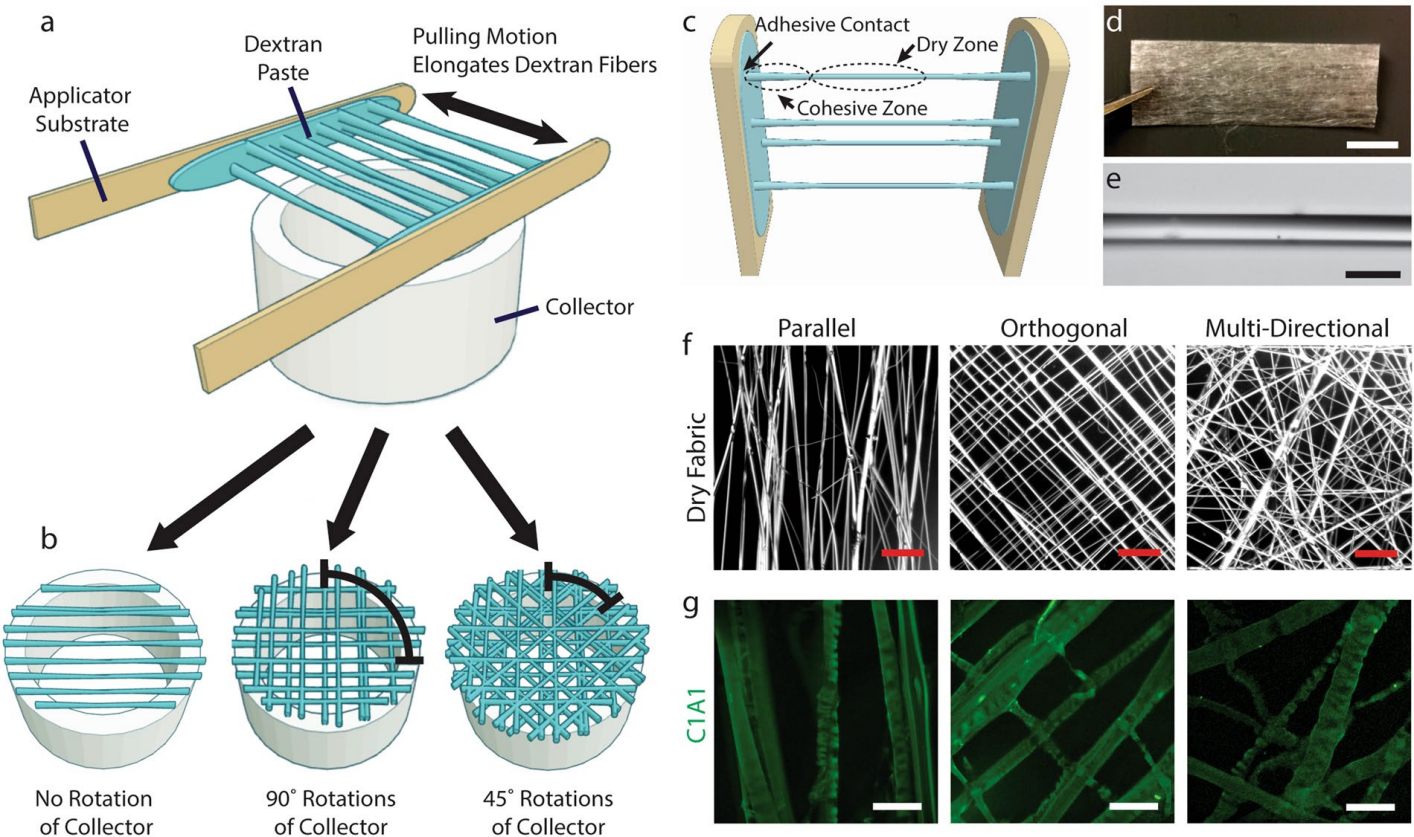
Dextran fabrics enable templated assembly of organized networks of collagen fibers. (**a**) Dextran fabrics are formed by repeated elongation of fibers from a viscous dextran paste pressed between two planar substrates. (**b**) Fiber orientation is controlled by rotation of a hollow collector that is used to support the ends of the fibers. (**c**) Adhesive forces between the dextran solution and the substrates balance with cohesive forces to extend the fibers as they dry in a central zone. (**d**) Dry fabrics are shelf-stable and can be easily manipulated for a variety of applications. Fabrics can also be trimmed to desired dimensions. Scale bar, 1 mm. (**e**) Single fibers are uniform and can be easily fabricated and collected. Shown is a region of a single fiber imaged by brightfield microscopy. Scale bar, 50 µm. (**f**) Fabrics can contain fibers in various configurations including parallel bundles, orthogonal lattices, and multi-directional lattice configurations, as shown in darkfield stereomicroscopy images. Scale bars, 500 µm. (**g**) Upon rehydration, dextran dissolves rapidly from collagen-doped fabrics, leaving behind a network of collagen fibers corresponding to the dry template configuration. Immunofluorescence images for C1A1, an antibody that recognizes native helical collagen, demonstrate appropriate collagen self-assembly. Scale bars, 50 µm.

Dextran fibers readily elongate as a viscous solution of 50% dextran is pressed and pulled apart between two substrates, e.g., two sterile tongue depressors (Fig. 1a–c). Fiber elongation is mediated by the balance of adhesive forces between the dextran solution and the two substrates and cohesive forces within the viscous dextran solution. Fibers elongate from numerous points between the two substrates, allowing multiple fibers to be generated at once. Dextran fibers rapidly dry as the fibers elongate from viscous cohesive regions proximal to the two substrates, but remain flexible enough for collection without breakage. This allows multiple layers of fibers to be applied to generate fabrics that are several hundred layers thick (Fig. 1d). Individual elongated dextran fibers have a smooth, glass-like appearance and are typically between 10 and 100 µm in diameter (Fig. 1e). The collection substrate can be rotated between fiber elongation cycles to generate fabrics with various fiber configurations, including fabrics containing parallel fibers, orthogonal fibers and multidirectional fibers (Fig. 1f). Dry fabrics stored under normal atmospheric conditions remain stable for several months without any noticeable loss of shape, fiber morphology or material properties.

Incorporation of Type I collagen into the dextran solution has no significant influence on the fiber formation process, as indicated by comparison of dry fiber diameter, dry fiber density (fibers/unit area) and dry pore size for collagen-doped and control fabrics (Supplementary Figure 1a–c). However, both the density and diameter of collagen fibers present after rehydration increase with increasing collagen mass fraction, while the pore size or area between fibers decrease (Supplementary Figure 1a–c). Upon rehydration in an appropriate buffer for collagen fiber assembly, the dextran rapidly dissolves (Supplementary Figure 2), leaving behind a network of collagen fibers (Fig. 1g). Fabrics containing collagen:dextran mass fractions of 0.004 and above efficiently form collagen-rich fibers upon rehydration (Supplementary Figure 3). Most collagen fibers range between 5 and 20 µm in diameter, approximating the size scale of native collagen fibers observed in various tissues. These collagen fibers are recognized by antibodies to the native (helical, non-denatured) form of Type I collagen, suggesting that our fiber manufacturing process produces fibers containing collagen molecules with appropriate secondary protein structures. Collagen fibers produced by this process also display intrinsic birefringence when imaged by polarized light microscopy (Supplementary Figure 4). This crystalline-like optical behavior indicates ordering of collagen molecules parallel to the fiber axis, which is one of the hallmarks of properly assembled collagen fibers^47^. In addition, collagen fibers stain strongly with aniline blue (Supplementary Figure 5), further suggesting that the structures that remain after dextran dissolution are primarily composed of collagen.

**Figure 2:**
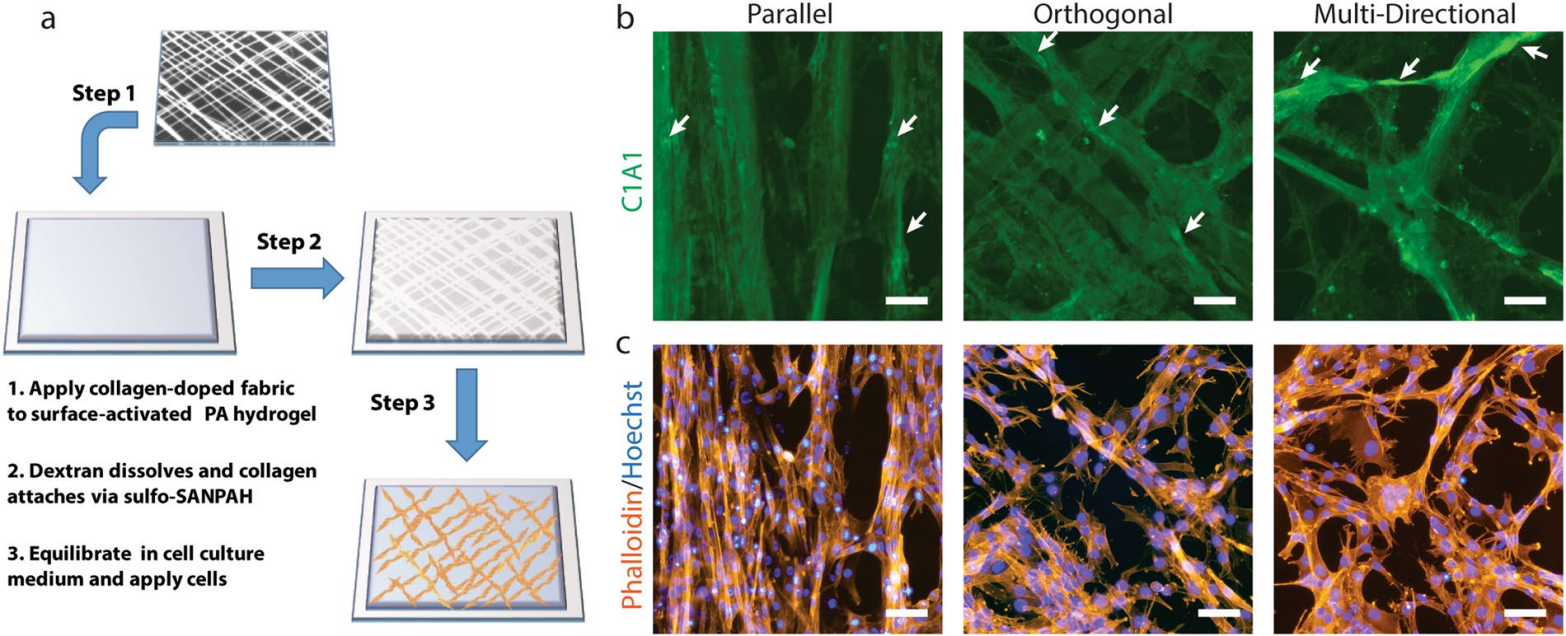
Hydrogel surface functionalization with collagen fiber networks directs skeletal muscle cell growth. (**a**) Collagen networks can be immobilized on the surface of polyacrylamide hydrogels to facilitate analysis of cell growth. (**b**) Collagen networks remain bound to the hydrogel surfaces following cell culture. Images shown are C1A1 immunofluorescence after 3 days in culture. Arrows indicate regions of additional cellular deposition of Type I collagen. (**c**) Actin-phalloidin and Hoechst nuclear staining of cells growing along the collagen fiber networks shown in (**b**). All scale bars, 50 µm.

The collagen fibers produced by our manufacturing process can be used as a surface-anchored biomaterial network capable of directing growth of C2C12 myoblasts (Fig. 2a). Attaching the collagen fibers as a 2D network to the surface of a non-adherent polyacrylamide hydrogel facilitates observation of C2C12 attachment, alignment and growth. Collagen fibers bound to the surface of non-adherent polyacrylamide hydrogels retain their fiber orientations during cell culture (Fig. 2b, Supplementary Figure 6). Over a 3-day period of cell culture, C2C12 cells selectively attach and grow along the fibers, as indicated by actin-phalloidin staining and Hoechst nuclear staining (Fig. 2c), forming fascicle-like structures. Inspection of cell growth patterns indicates that both the cells and the actin cytoskeleton within the cells align with the collagen network. In addition, undifferentiated C2C12 cells gradually remodel the collagen network by displacing collagen fibers through traction force and by synthesizing and secreting additional Type I collagen along existing fibers (arrows in Fig. 2b).

**Figure 3:**
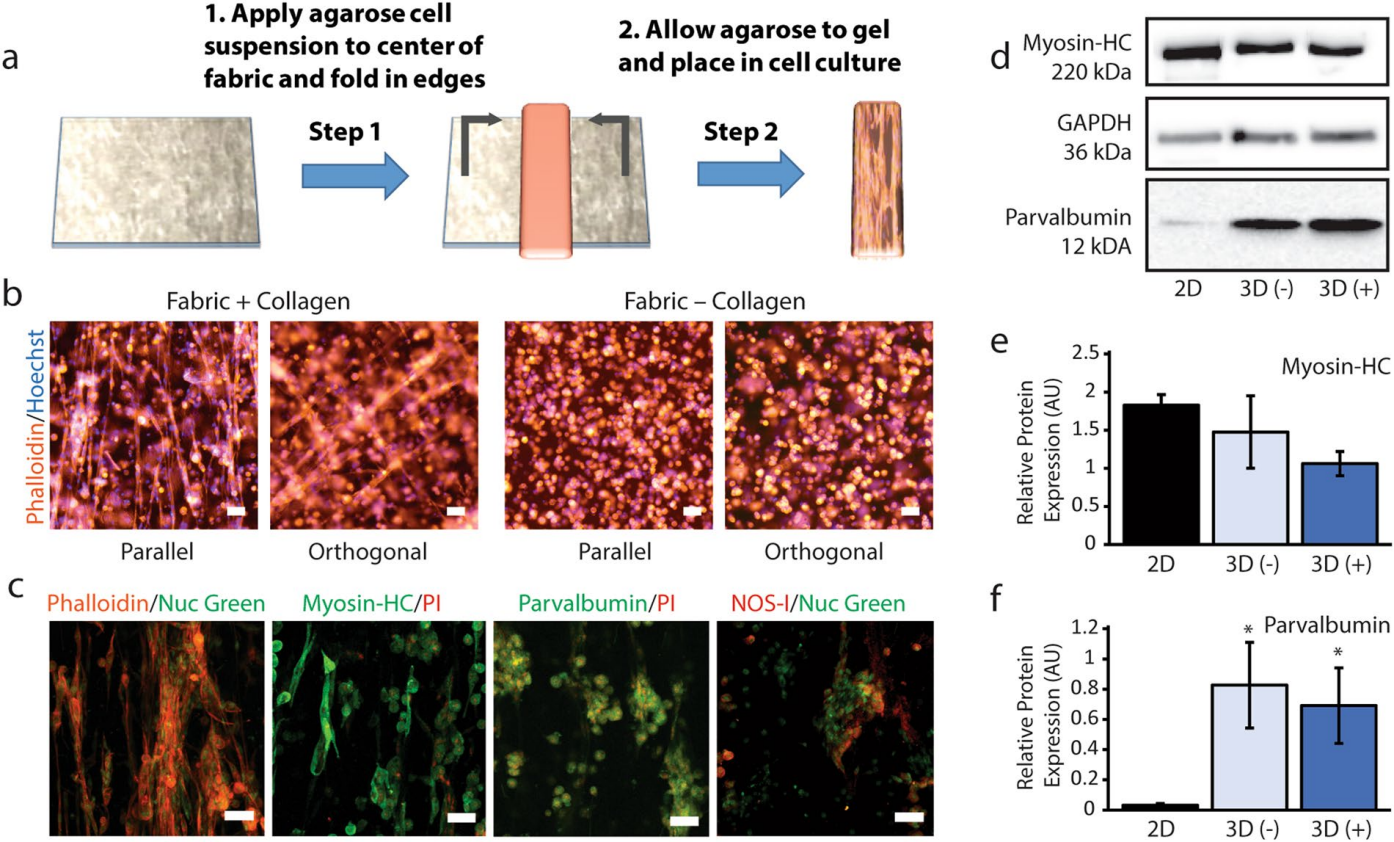
Incorporation of collagen networks in 3D hydrogel scaffolds supports cell alignment and differentiation. (**a**) Dry fabrics can be readily incorporated into commonly-used non-cell-adherent hydrogels such as agarose. (**b**) Low magnification epifluorescence images of actin-phalloidin and Hoechst nuclear staining demonstrate that collagen-doped fabrics promote large-scale alignment of agarose-encapsulated C2C12 cells. Cell alignment and growth is not observed for control fabrics that do not contain collagen. Arrows indicate direction of fabric alignment. (**c**) High-resolution confocal maximum intensity projections show details of aligned fascicle-like structures. Positioning of cell nuclei and interconnected cytoskeleton structures are indicative of myotube formation. Myotubes and fascicle-like structures are not observed for cells cultured with control fabrics. C2C12 cells growing along collagen networks within the hydrogels display immunofluorescence for skeletal muscle cell differentiation markers including myosin heavy chain, parvalbumin and NOS-I. All scale bars, 50 µm. (**d**) Representative Western blots for myosin heavy chain, parvalbumin and GAPDH for cells grown in 2D on tissue culture plastic, within agarose containing 3D control fabrics (3D (−)) and within agarose containing 3D collagen fabrics (3D (+)). Images are cropped from the original full blots with adjustment of brightness and contrast to ensure that faint bands are visible. Unprocessed Western blot images are available in Supplementary Figure 7. Levels of myosin heavy chain (**e**) and parvalbumin (**f**) relative to GAPDH (AU; Arbitrary Units) were compared across culture conditions. Significant differences of p < 0.05 by one-way ANOVA on ranks and Dunnett’s multiple comparison test with respect to the 2D control group are indicated by ^*^. Densitometry data are available in Supplementary Table 1.

Collagen-doped fabrics are also capable of aligning cells grown in a 3D environment (Fig. 3a). After 7 days of 3D cell culture in low gelling point agarose hydrogels containing collagen-doped unidirectional fabrics, numerous elongated cells are evident by actin-phalloidin staining (Fig. 3b). In contrast, cells growing in agarose surrounding control fabrics (no collagen) remain clustered in spherical structures, indicating a lack of cell attachment and growth within the hydrogel. High-resolution confocal imaging indicates that aligned structures form from cells growing in continuous chain-like structures, indicating the potential for myotube formation as the cells differentiate (Fig. 3c). Myotube-like structures were not observed in agarose hydrogels containing control fabrics without collagen. Many of the aligned cells displayed markers of differentiated skeletal muscle, as indicated by staining of subpopulations of elongated cells growing on collagen fibers for myosin heavy chain (a motor protein found in skeletal muscle thick filaments), parvalbumin (a high-affinity Ca^2+^-binding protein found in fast-contracting skeletal muscle fibers), and NOS-I (the nitric oxide synthase isoform associated with the sarcolemma of skeletal muscle) (Fig. 3c).

To compare differences in expression of differentiation markers between 2D and 3D cultures conditions, we performed Western blot analysis on whole-cell lysates collected from differentiated cells (Fig. 3d–f, Supplementary Figure 7 and Supplementary Table 1). Relative myosin heavy chain expression was not significantly different across 2D and 3D culture conditions (Fig. 3e); however, cells grown in 3D matrices displayed significantly higher relative expression of parvalbumin (Fig. 3f). These data suggest that although cells growing under both 2D and 3D conditions can be induced to differentiate in response to appropriate biochemical treatment, there may be subtle differences in the expression of markers associated with skeletal muscle between 2D and 3D conditions. Notably, although differentiation marker expression was similar in 3D conditions with and without collagen fibers, only cells growing in scaffolds containing collagen formed elongated myotubes similar to C2C12 cells differentiated under 2D conditions. Collectively, these results demonstrate that our method for forming 3D collagen networks can support the growth and differentiation of skeletal muscle cells within non-cell-adherent hydrogels.

## Discussion

Using C2C12 cells, which are a well-established model cell line for understanding skeletal muscle development and physiology, we demonstrate production and application of a novel collagen-based biomaterial capable of organizing muscle cells into fascicle-like structures. In muscle tissue, fascicles are present in various patterns, giving rise to unique force generation properties within the tissue. Within each fascicle, groups of muscle cells are organized by collagen fibers, which can extend along or across muscle cells, before eventually inserting into tendons^48^. The novel collagen-based biomaterial characterized in this study is especially well suited for fabricating tissue models that display this pattern of collagen fiber alignment and cell organization.

Here, we demonstrate that collagen-doped fabrics can be used to support highly-specific myoblast growth in conjunction with two types of non-cell-adherent hydrogels (i.e., poly-acrylamide and agarose). One of the most attractive features of our material is that it allows cells encapsulated within hydrogels to elongate and grow into networks. This is one of the most challenging aspects of 3D cell culture that limits the applicability of many hydrogel systems as *in vitro* models and as materials for regenerative medicine. For example, many biological scaffolds that are naturally cell-adherent are also poorly defined in terms of their compositions, while many synthetic polymer-based hydrogels require costly and difficult to perform functionalization procedures to render them cell-adherent. In addition, to align cells in one or more direction, a background material that limits cell adhesion is preferred. Since the collagen network produced by our manufacturing process is a free-standing structure, it is highly versatile and can be applied to functionalize a variety of hydrogel scaffolds and surfaces, e.g., other non-adherent hydrogels such as alginate or polyethylene glycol diacrylate. Furthermore, this material has the potential to be used for large-scale network fabrication of organized skeletal muscle cells within a variety of natural and synthetic biomaterials for modeling musculoskeletal disease. In addition to myotube formation in skeletal muscle, various other cell types such as neural cells and vascular cells depend on network formation for appropriate physiological function. Since collagen has been demonstrated to support the growth of these and many other cell types, our approach may prove beneficial to constructing a wide range of model tissues^49^.

Future work will be conducted to examine the degradability and mechanical properties of the collagen-doped fabrics and hydrated collagen networks, e.g., their tensile strength and elastic modulus, as well as to determine if similar fiber structures can be formed from other materials, e.g. gelatin, fibrin or other types of extracellular matrix constituents. If materials with alternative biomechanical and biodegradation characteristics are desired, collagen and synthetic and/or natural material composites could also be synthesized^49^. For instance, polycaprolactone/collagen fabrics generated using electrospinning have been previously observed to have excellent mechanical properties and are increasingly being used for a variety of biomaterial applications^50^. Our technique could potentially improve upon the macro- and micro-structural organization and functionality of these collagen-based composite materials, while simultaneously minimizing production complications and cost. Bioactive agents could also be included in our fiber constructs, which would allow for controlled elution of useful drugs and/or biomolecules in addition to delivery of a collagen matrix to augment cell attachment, alignment and growth. While here we focus mainly on generating pure collagen biomaterials to functionalize 2D and 3D cell culture substrates, our previous work with drug-eluting dextran fabrics could be combined with our most recent findings to address wound healing applications. For example, we previously observed that dextran fabrics containing thrombin and antibiotics effectively promoted coagulation of human platelet poor plasma and suppressed bacterial growth *in vitro* ^46^. The addition of collagen fibers could further promote endothelial migration, fibroblast proliferation and platelet aggregation to enhance wound healing with this material.

In terms of manufacturing, one of the strengths of our collagen fiber formation process is that it only requires simple motions to form elongated fibers. Thus, the overall procedure can be adapted to an automated manufacturing system to further improve throughput and consistency. For example, programmed volumes of collagen-doped dextran could be extruded onto two flat arms. Pressing and releasing the two arms to a set distance along the y-axis would induce fiber formation. As the fibers form, the arms could then travel down the z-axis towards a collector where the fibers would be deposited. A turning motion of the collector with a partial lip on both ends could then clip the ends of the fibers, and the arms would return to their initial positioning, where additional collagen-doped dextran could be added and the process repeated for a predetermined number of layers. Fiber orientation could be controlled with programmed turns of the collector to generate consistently organized fabrics with defined fiber directions. In addition, it would be possible to adjust the pore size between fibers by increasing or decreasing the density of fibers in the material. Alternatively, it may be possible to develop substrates with surface features designed to control the location at which fibers form during each elongation cycle for improved control over pore size. An automated manufacturing scheme such as this, utilizing standard robotic designs and simple movements, would enable mass manufacturing of our materials for healthcare applications. An additional strength of our approach is that it utilizes only a few reagents that are already produced in cGMP facilities and are readily available as pharmaceutical grade preparations.

## Conclusion

In conclusion, we present an entirely new process for fabricating collagen fibers of appropriate size scales for promoting interaction with cells. We demonstrate that the fibers produced by our manufacturing process can be aligned in several configurations and that the collagen from which the fibers are formed displays the appropriate molecular organization (e.g., intact α-helical structure and birefringence). We also demonstrate that myoblasts readily attach and align along 2D collagen fiber networks. Furthermore, encapsulation of collagen fibers with cells into non-cell-adherent hydrogels promotes aligned growth of cells and supports their differentiation. The ease-of-production and versatility of this novel collagen-based biomaterial will support future development of a variety of tissue-engineered disease models and materials for regenerative medicine and wound reconstruction.

## Methods

### Materials and Reagents

Building block dextran (molecular weight 500,000 g/mol) from Dextran Products Ltd. (Scarborough, ON Canada) was used for optimization of the fiber formation process. Based on the manufacturer’s certification tests, the specific rotation (2 wt.% solution, ${[\alpha ]}_{D}^{20}$) was +195° and the intrinsic viscosity (1 wt. % solution, 37 °C) was 0.58 dL/g. Dextran fabrics used in cell culture experiments were formed using high-purity technical quality dextran (molecular weight 500,000 g/mol) from Pharmacosmos (Holbaek, Denmark). Collagen Type I (Rat Tail, High Concentration) was purchased from Corning (Corning, NY USA). All other chemicals were reagent grade or better and were used without further modification or purification.

### Dextran Fabric Formation

Dextran fabrics were formed as described previously^46^. Briefly, a 50 wt.% solution of dextran was produced by blending dry dextran with ultrapure water using a pipette tip to produce a viscous paste. A thin layer of dextran paste was then applied to two sterile tongue depressor sticks. The sticks were repeatedly pressed together and pulled apart such that fibers of highly-viscous dextran elongated between the two sticks. After each cycle of pressing and pulling, the dry elongated fibers were collected over the top of a 5.5 cm diameter beaker (Fig. 1a,b). Repeated cycles yielded dense, multilayer fabrics that could be trimmed for removal from the beaker and stored dry at room temperature. To produce collagen-doped fabrics, collagen was dissolved in ultrapure water prior to mixing with dextran to achieve final mass fractions of collagen in the dry material of 0.001, 0.002, 0.004, 0.006 and 0.008. Upon re-hydration in DMEM or an appropriate buffer for collagen fiber self-assembly^51^, the dextran template rapidly dissolved leaving behind networks of collagen fibers of the same geometry as the original fabric.

### Cell Culture

C2C12 mouse myoblasts (ATCC, CRL-1772) were cultured per ATCC instructions. Briefly, cells were cultured in growth medium consisting of DMEM containing 1% antibiotic antimycotic solution and 10% fetal bovine serum. Cells were passaged at ~50–60% confluence and used before 20 passages. For differentiation experiments, C2C12 cell were cultured in differentiation medium consisting of DMEM containing 1% antibiotic antimycotic solution, 2% fetal bovine serum, and 1% insulin, transferrin and selenium (ITS) solution. All cell cultures were maintained in a humidified incubator at 37 °C under 5% CO_2_.

### 2D Polyacrylamide Hydrogel Fabrication

Polyacrylamide hydrogels for immobilizing 2D networks of collagen fibers were fabricated by modifying existing procedures^52^. Briefly, glass coverslips (22 × 22 mm) were placed on a 95 °C hotplate and covered with 500 µL 0.1 N NaOH to activate the surface. The NaOH was evaporated and 500 µL of ddH_2_0 was then applied and evaporated to improve the uniformity of the surface coating. The NaOH-coated coverslips were then treated with a 4% solution of (3-Aminopropyl)triethoxysilane in acetone for 15 min at room temperature. The resulting amine-functionalized coverslips were washed three times in ddH_2_0 and air-dried. The coverslips were then exposed to a 0.8% solution of glutaraldehyde in PBS for 1 hour at room temperature. The coverslips were again washed three times in ddH_2_0 and air-dried. Glutaraldehyde provided bi-functional cross-linking of the surface amines on the coverslips to the amide groups of the acrylamide-based hydrogels. To form uniform polyacrylamide hydrogels (shear modulus ~8.64)^52^ on the surface of the coverslips, solutions consisting of 7.5% acrylamide, 0.3% bis-acrylamide, 0.08% ammonium persulfate and 0.001% TEMED were sandwiched between the glutaraldehyde-coated coverslips (binding) and glass slides treated with trichlorosilane (release). After 30 minutes of polymerization, the coverslips containing polyacrylamide hydrogels were carefully removed from the slides and washed three times in ddH_2_0. To provide sites for attachment of collagen fibers, the hydrogel surfaces were functionalized by incubation in a 2 mg/mL solution of sulfo-SANPAH under UV light for 5 minutes. The coverslips were washed five times in ddH_2_0 and a final time in DMEM. Finally, the DMEM was completely removed and dry fabrics (10 layers thick) were applied to the surfaces to allow the collagen fibers to attach to the hydrogels. After 1 hour of incubation, the hydrogels containing surface-bound collagen fibers were placed in sterile 6-well culture plates and prepared for cell culture by incubation in growth medium. Approximately, 0.5 × 10^6^ cells were added to each well. After incubation in growth medium for 3 days, the samples were fixed in 3.7% formalin for 15 minutes at room temperature for subsequent immunolabeling of collagen and Hoechst/Phalloidin staining. A total of 3 independent experiments were performed with triplicate samples for each condition within each experiment.

### 3D Agarose Hydrogel Fabrication

Agarose gels were formed by dissolving SeaPlaque^TM^ Agarose (Lonza, Rockland, ME USA) in serum-free culture medium to a concentration of 2 wt.% at temperatures above 70 °C. The agarose solution was held at 37 °C in a water bath during cell preparation. An equal volume of warm cell suspension (20 × 10^6^ cells/mL) and 2% agarose were mixed to achieve a final cell concentration of 10 × 10^6^ cells/mL and a final agarose concentration of 1%. A volume of 75 µL of this mixture was immediately pipetted onto each ~15 mg dextran-fabric contained within separate wells of a 6-well culture plate. After 5 minutes of gelation, 2 mL of growth medium was added to each well. The following day, the growth medium was replaced with differentiation medium and the cells were cultured for an additional 6 days. The differentiation medium was replenished on days 3 and 5. On day 7, the samples were either lysed in NP-40 buffer containing SIGMAFAST protease inhibitor for Western blot analysis or fixed in 3.7% formalin for 30 minutes at room temperature for subsequent immunolabeling and staining. A minimum of 3 independent experiments were analyzed by immunolabeling and Western blot.

### Immunolabeling, Staining and Microscopy

A mouse-anti-collagen I alpha 1 antibody (1:100; Novus Biologics, Oakville, ON Canada) to the native (helical form) of Type I Collagen was used to label properly-assembled collagen structures in 2D conditions. The following primary antibodies were used to assess C2C12 differentiation in 3D culture conditions by immunofluorescence: mouse-anti-myosin-hc (1:100; Novus Biologicals), mouse-anti-parvalbumin (1:200; Chemicon), rabbit-anti-parvalbumin (1:100; Novus Biologicals), rabbit-anti-NOS-I (1:200; Chemicon) and goat-anti-nNOS (NOS-I) (1:100; Novus Biologicals). Two different antibodies were used to confirm appropriate staining for parvalbumin and NOS-I in C2C12 cells due to the diffuse expression of these markers in the cytoplasm. HyLite 488-conjugated anti-mouse IgG (Novus Biologicals), HyLite 555-conjugated anti-rabbit IgG (Novus Biologicals) and DyLight 488-conjugated anti-goat IgG (Novus Biologicals) secondary antibodies were used for immunofluorescence detection as appropriate. Prior to immunolabeling of differentiated cells, formalin-fixed samples were permeabilized in 0.5% Triton X-100 for 30 minutes and subsequently blocked in 2% bovine serum albumin (BSA) for 2 hours. The samples were then incubated in primary antibodies overnight at 4 °C, washed 5 times for 30 min per wash, incubated in secondary antibodies overnight at 4 °C and washed again 5 times for 30 min per wash.

Rhodamine-conjugated phalloidin was used to assess actin cytoskeleton organization for various culture conditions. Hoechst 33342 (Sigma Aldrich, St. Louis, MO), propidium iodide (Sigma) and Nuclear Green DCS1 (Abcam, Toronto, ON Canada) were used for nuclear counterstaining as indicated. Images were acquired using a Nikon Eclipse Ti epifluorescence microscope for 2D cell cultures and low magnification images of 3D cell cultures. A Zeiss LSM 510 Meta confocal system was used to acquire high resolution images of 3D cell cultures. Stereomicroscopy was used to acquire images of dry fabrics. A Nikon polarizing microscope was used to acquire images of collagen fiber birefringence.

### Western Blot Analysis

Whole cell lysate protein concentrations were measured by Bradford assay to ensure equal protein loading. Proteins were separated by polyacrylamide gel electrophoresis using 4–20% gradient gels. Proteins were then transferred to 0.2 µm pore size polyvinylidene fluoride membranes. The membranes were blocked in 5% BSA in tris-buffered saline buffer containing Tween 20 (TBST) for 1.5 hours. The membranes were then incubated in primary antibody solutions containing 1% BSA in TBST overnight at 4 °C. Following primary antibody incubation, the membranes were washed 3 times for 10 minute each wash in TBST. After washing, the membranes were incubated in secondary antibody solutions containing 1% BSA in TBST for 1 hour. SuperSignal West Pico chemiluminescence substrate was used for detection. Images were recorded on an Azure C300 system using automatic exposure settings to prevent oversaturation of bands. The following primary and secondary antibodies were used: mouse anti-GAPDH (1:20,000; Novus Biologicals), mouse-anti-Myosin-hc (1:250; Novus Biologicals), rabbit-anti-parvalbumin (1:10,000; Novus Biologicals), horseradish peroxidase (HRP)-conjugated goat-anti-mouse IgG (1:1000; Novus Biologicals) and HRP-conjugated goat-anti-rabbit IgG (1:1000; Novus Biologicals). Densitometry was performed in ImageJ using the Gel Analysis tool.

## Electronic supplementary material

Supplementary Information

## References

[1] Gelse K, Pöschl E, Aigner T (2003). Collagens—structure, function, and biosynthesis. Advanced Drug Delivery Reviews.

[2] Ricard-Blum S (2011). The collagen family. Cold Spring Harbor perspectives in biology.

[3] Soderhall C (2007). Variants in a novel epidermal collagen gene (COL29A1) are associated with atopic dermatitis. PLoS biology.

[4] Shoulders MD, Raines RT (2009). Collagen structure and stability. Annual review of biochemistry.

[5] Svensson RB, Mulder H, Kovanen V, Magnusson SP (2013). Fracture mechanics of collagen fibrils: influence of natural cross-links. Biophysical journal.

[6] Depalle B, Qin Z, Shefelbine SJ, Buehler MJ (2015). Influence of cross-link structure, density and mechanical properties in the mesoscale deformation mechanisms of collagen fibrils. Journal of the Mechanical Behavior of Biomedical Materials.

[7] de Wild M, Pomp W, Koenderink GH (2013). Thermal memory in self-assembled collagen fibril networks. Biophysical journal.

[8] Ushiki T (2002). Collagen fibers, reticular fibers and elastic fibers. A comprehensive understanding from a morphological viewpoint. Archives of histology and cytology.

[9] Hulmes DJS (2002). Building Collagen Molecules, Fibrils, and Suprafibrillar Structures. Journal of Structural Biology.

[10] Weis SM (2000). Myocardial mechanics and collagen structure in the osteogenesis imperfecta murine (oim). Circulation research.

[11] Liu, S. H., Yang, R. S., al-Shaikh, R. & Lane, J. M. Collagen in tendon, ligament, and bone healing. A current review. *Clinical orthopaedics and related research*, 265–278 (1995). 7671527

[12] Lovell CR (1987). Type I and III collagen content and fibre distribution in normal human skin during ageing. The British journal of dermatology.

[13] Huijing PA (1999). Muscle as a collagen fiber reinforced composite: a review of force transmission in muscle and whole limb. Journal of Biomechanics.

[14] Liu W (2008). Recombinant human collagen for tissue engineered corneal substitutes. Biomaterials.

[15] Toman PD (2000). Production of recombinant human type I procollagen trimers using a four-gene expression system in the yeast Saccharomyces cerevisiae. The Journal of biological chemistry.

[16] Ruggiero F (2000). Triple helix assembly and processing of human collagen produced in transgenic tobacco plants. FEBS letters.

[17] Zeugolis DI, Paul RG, Attenburrow G (2008). Factors influencing the properties of reconstituted collagen fibers prior to self-assembly: animal species and collagen extraction method. Journal of biomedical materials research. Part A.

[18] Santos MH (2013). Extraction and characterization of highly purified collagen from bovine pericardium for potential bioengineering applications. Materials science & engineering. C, Materials for biological applications.

[19] Heino J (2000). The collagen receptor integrins have distinct ligand recognition and signaling functions. Matrix biology: journal of the International Society for Matrix Biology.

[20] Jokinen J (2004). Integrin-mediated cell adhesion to type I collagen fibrils. The Journal of biological chemistry.

[21] Hotary K, Allen E, Punturieri A, Yana I, Weiss SJ (2000). Regulation of cell invasion and morphogenesis in a three-dimensional type I collagen matrix by membrane-type matrix metalloproteinases 1, 2, and 3. The Journal of cell biology.

[22] Chattopadhyay S, Raines RT (2014). Review collagen-based biomaterials for wound healing. Biopolymers.

[23] Chevallay B, Herbage D (2000). Collagen-based biomaterials as 3D scaffold for cell cultures: applications for tissue engineering and gene therapy. Medical & biological engineering & computing.

[24] Nunes SS (2013). Biowire: a platform for maturation of human pluripotent stem cell-derived cardiomyocytes. Nature methods.

[25] Norman JJ, Desai TA (2005). Control of cellular organization in three dimensions using a microfabricated polydimethylsiloxane-collagen composite tissue scaffold. Tissue engineering.

[26] Chaubaroux C (2015). Cell Alignment Driven by Mechanically Induced Collagen Fiber Alignment in Collagen/Alginate Coatings. Tissue engineering. Part C, Methods.

[27] Vader D, Kabla A, Weitz D, Mahadevan L (2009). Strain-induced alignment in collagen gels. PloS one.

[28] Lee P, Lin R, Moon J, Lee LP (2006). Microfluidic alignment of collagen fibers for *in vitro* cell culture. Biomedical microdevices.

[29] Guo C, Kaufman LJ (2007). Flow and magnetic field induced collagen alignment. Biomaterials.

[30] Paten JA (2016). Flow-Induced Crystallization of Collagen: A Potentially Critical Mechanism in Early Tissue Formation. ACS Nano.

[31] Eguchi Y, Ohtori S, Sekino M, Ueno S (2015). Effectiveness of magnetically aligned collagen for neural regeneration *in vitro* and *in vivo*. Bioelectromagnetics.

[32] Hirose H, Nakahara T, Miyakoshi J (2003). Orientation of human glioblastoma cells embedded in type I collagen, caused by exposure to a 10 T static magnetic field. Neuroscience letters.

[33] Caliari SR, Harley BA (2011). The effect of anisotropic collagen-GAG scaffolds and growth factor supplementation on tendon cell recruitment, alignment, and metabolic activity. Biomaterials.

[34] Kroehne V (2008). Use of a novel collagen matrix with oriented pore structure for muscle cell differentiation in cell culture and in grafts. Journal of Cellular and Molecular Medicine.

[35] Abbah SA (2015). Harnessing Hierarchical Nano- and Micro-Fabrication Technologies for Musculoskeletal Tissue Engineering. Advanced healthcare materials.

[36] Huang Z-M, Zhang YZ, Kotaki M, Ramakrishna S (2003). A review on polymer nanofibers by electrospinning and their applications in nanocomposites. Composites Science and Technology.

[37] Matthews JA, Wnek GE, Simpson DG, Bowlin GL (2002). Electrospinning of Collagen Nanofibers. Biomacromolecules.

[38] Phipps MC, Clem WC, Grunda JM, Clines GA, Bellis SL (2012). Increasing the pore sizes of bone-mimetic electrospun scaffolds comprised of polycaprolactone, collagen I and hydroxyapatite to enhance cell infiltration. Biomaterials.

[39] Teo WE, Ramakrishna S (2005). Electrospun fibre bundle made of aligned nanofibres over two fixed points. Nanotechnology.

[40] Huang GP (2015). An investigation of common crosslinking agents on the stability of electrospun collagen scaffolds. Journal of biomedical materials research. Part A.

[41] Zeugolis DI (2008). Electro-spinning of pure collagen nano-fibres – Just an expensive way to make gelatin?. Biomaterials.

[42] Bürck J (2013). Resemblance of Electrospun Collagen Nanofibers to Their Native Structure. Langmuir.

[43] Caves JM (2010). Fibrillogenesis in continuously spun synthetic collagen fiber. Journal of biomedical materials research. Part B, Applied biomaterials.

[44] Haynl C, Hofmann E, Pawar K, Förster S, Scheibel T (2016). Microfluidics-Produced Collagen Fibers Show Extraordinary Mechanical Properties. Nano letters.

[45] Gillette BM (2008). *In situ* collagen assembly for integrating microfabricated three-dimensional cell-seeded matrices. Nature materials.

[46] Frampton JP (2015). Elongation of fibers from highly viscous dextran solutions enables fabrication of rapidly dissolving drug carrying fabrics. Advanced healthcare materials.

[47] Giraud-Guille MM, Besseau L, Martin R (2003). Liquid crystalline assemblies of collagen in bone and *in vitro* systems. J Biomech.

[48] Gillies AR, Lieber RL (2011). Structure and function of the skeletal muscle extracellular matrix. Muscle & nerve.

[49] Walters BD, Stegemann JP (2014). Strategies for directing the structure and function of three-dimensional collagen biomaterials across length scales. Acta biomaterialia.

[50] Tillman BW (2009). The *in vivo* stability of electrospun polycaprolactone-collagen scaffolds in vascular reconstruction. Biomaterials.

[51] Pins GD, Christiansen DL, Patel R, Silver FH (1997). Self-assembly of collagen fibers. Influence of fibrillar alignment and decorin on mechanical properties. Biophysical journal.

[52] Fischer RS, Myers KA, Gardel ML, Waterman CM (2012). Stiffness-controlled three-dimensional extracellular matrices for high-resolution imaging of cell behavior. Nature protocols.

