# Supplementary material for: Templated Assembly of Collagen Fibers Directs Cell Growth in 2D and 3D
Source: Sci Rep. 2017 Aug 29;7:9628. doi: 10.1038/s41598-017-10182-8 (PMC5575125; doi:10.1038/s41598-017-10182-8)
Supplement: Supplementary file 1 — Supplementary Information [file 41598_2017_10182_MOESM1_ESM.pdf]

Supplementary Information

Templated Assembly of Collagen Fibers Directs Cell Growth in 2D and 3D

G.Y. Liu[1], R. Agarwal[1], K.R Ko[1], M. Ruthven[1], H.T. Sarhan[1], and J.P. Frampton[1]

[1]School of Biomedical Engineering, Dalhousie University, Halifax, Nova Scotia Canada

To whom correspondence should be addressed:

John P. Frampton, PhD

School of Biomedical Engineering

Dalhousie University

Halifax, NS, Canada B3H 4R2

Email: john.frampton@dal.ca

Phone: 902-494-4175

Supplementary Methods

Aniline Blue Staining

Formalin-fixed collagen networks were incubated in a solution of 2.5% phosphotungstic acid/2.5% phosphomolybdic acid in distilled water for 10 minutes. The collagen networks were then stained using a 2.5% solution of aniline blue in 1% acetic acid for 5 minutes. The collagen networks were rinsed in distilled water, cleared in 1% acetic acid, and rinsed again in distilled water before imaging by brightfield microscopy.

Image Analysis

Fiber diameter, density (number of fiber/ROI) and pore size of dry and hydrated collagen fabrics were determined by analyzing stereomicroscope images using Fiji. A 1.2 mm x 1.2 mm region of interest (ROI) was selected at the center of each image. Wet and dry fiber diameters, densities and pore sizes within the ROIs were measured. Mean fiber density and diameter ± the standard error of the mean were graphed as a function of collagen mass fraction. For analysis of fiber orientation, thresholded images of dry fabrics and C1A1 immunofluorescence were processed using the OrientationJ plugin for Fiji[1].

[1]Z. Püspöki, M. Storath, D. Sage, M. Unser, "Transforms and Operators for Directional Bioimage Analysis: A Survey," *Advances in Anatomy, Embryology and Cell Biology*, vol. 219, Focus on Bio-Image Informatics, Springer International Publishing, ch. 3, pp. 69-93, May 21, 2016.

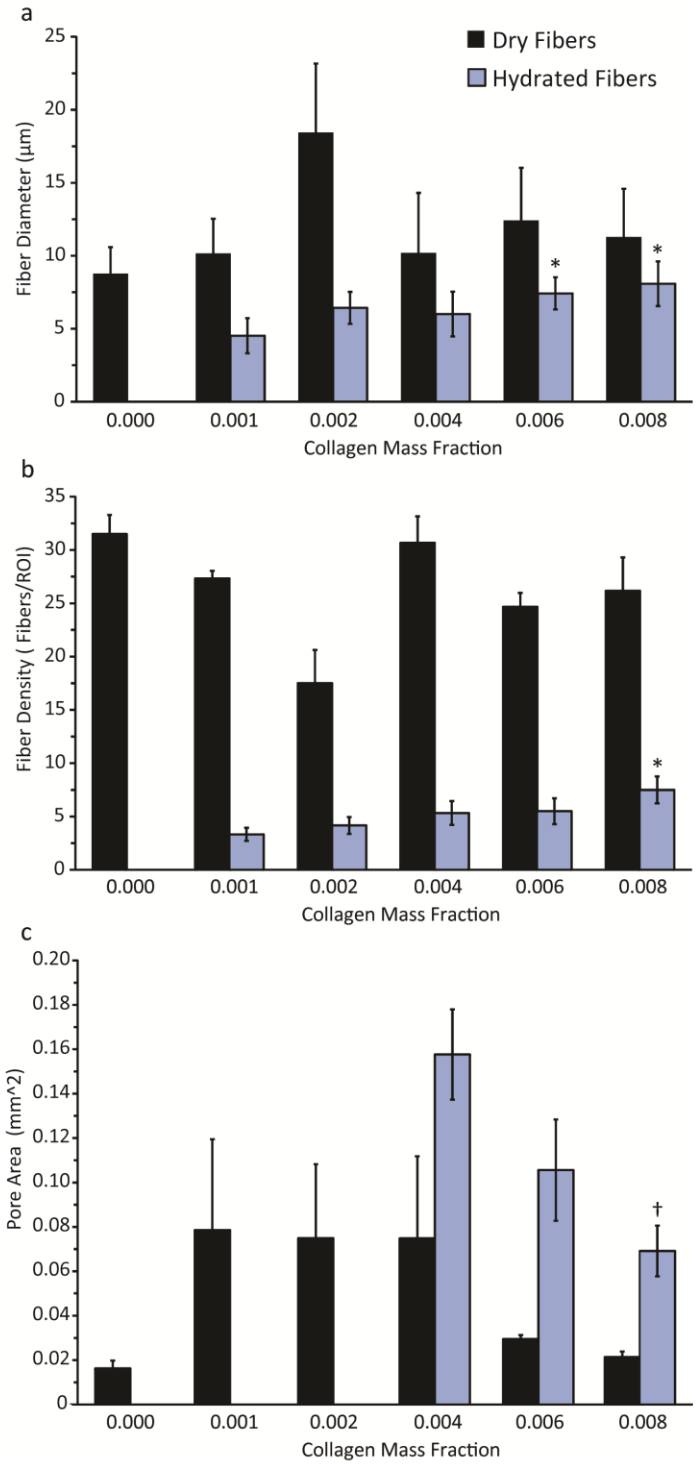
a
Dry Fibers
Hydrated Fibers
Fiber Diameter (µm)
25
20
15
10
5
0
0.000
0.001
0.002
0.004
0.006
0.008
Collagen Mass Fraction
b
Fiber Density (Fibers/ROI)
35
30
25
20
15
10
5
0
0.000
0.001
0.002
0.004
0.006
0.008
Collagen Mass Fraction
c
Pore Area (mm^2)
0.20
0.18
0.16
0.14
0.12
0.10
0.08
0.06
0.04
0.02
0
0.000
0.001
0.002
0.004
0.006
0.008
Collagen Mass Fraction

Supplementary Figure 1. Analysis of fibers before and after hydration. (a) Hydrated collagen fiber diameter increases slightly with increasing collagen mass fraction. **(b)** The density of hydrated fibers (number of fibers per ROI; ROI=1.45 mm^2) increases with increasing collagen mass fraction. **(c)** The pore size of the hydrated material decreases with increasing collagen mass fraction, presumably due to the higher density of fibers that remain upon rehydration. Pore size could not be calculated for the 0.001 and 0.002 samples due to the low numbers of fibers that formed under these conditions. Fabrics without collagen do not produce fibers. Data are presented as mean values ± the standard error of the mean. Significant differences from the 0.001 mass fraction for hydrated fibers are indicated by *. Significant differences from the 0.004 mass fraction for hydrated fibers are indicated by †. Significant differences were observed for fiber diameter, density and pore size between dry and hydrated fibers. One-way ANOVA with the Tukey post-hoc test was used to determine statistical significance within the dry and hydrated groups. Two-way ANOVA was used to determine statistical significance between the dry and hydrated groups. $p<0.05$ was considered significant.

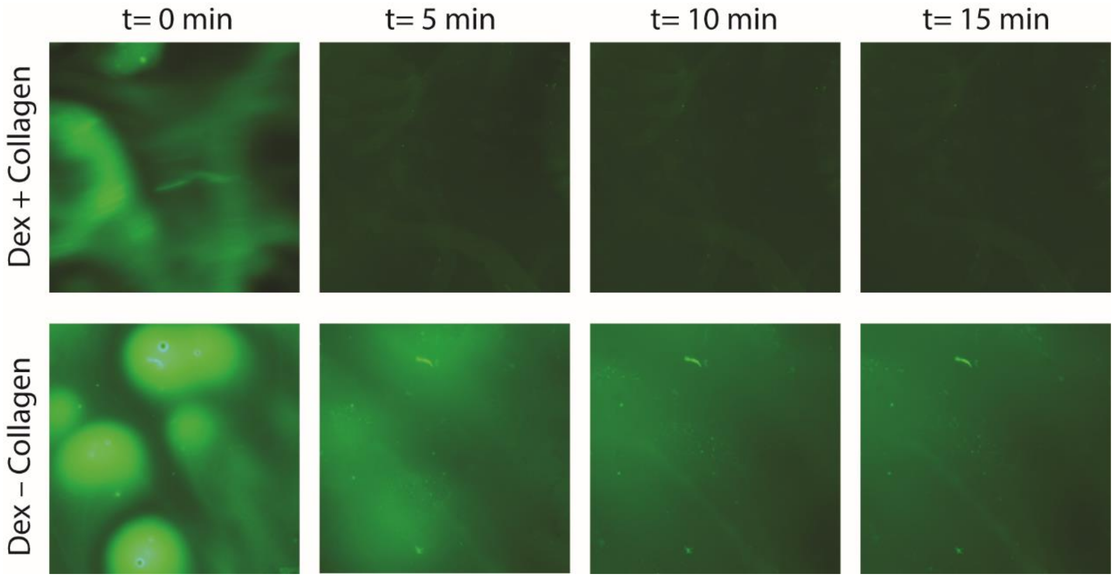

Supplementary Figure 2. Dextran dissolves rapidly following fabric hydration. Dextran completely dissolves in the liquid medium over approximately 15 minutes for both control fabrics (no collagen) and collagen-doped dextran fabrics.

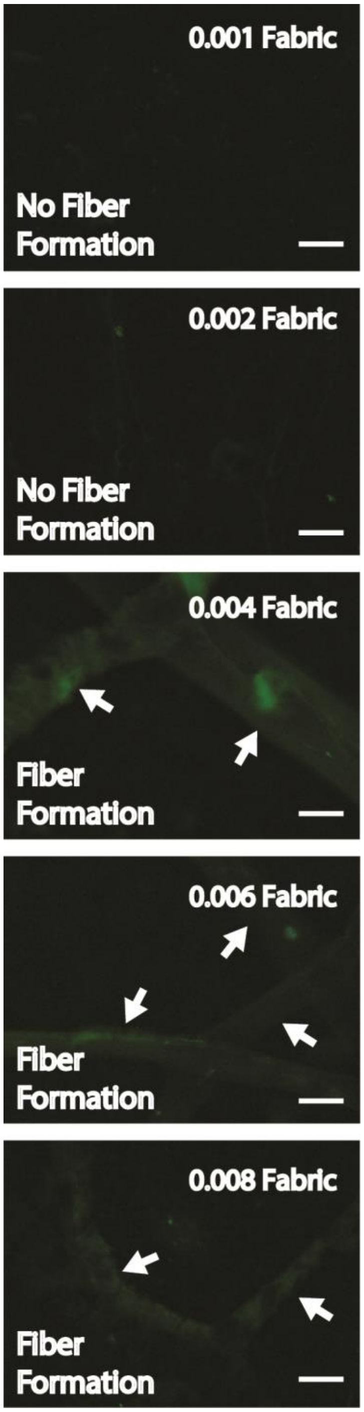

Supplementary Figure 3. Effect of collagen mass fraction on collagen fiber integrity. Mass fractions of collagen of 0.004 or greater form robust collagen fiber networks that display C1A1immunofluorescence. Delicate fibers form sporadically for lower mass fractions of collagen. Scale, 20 μm. Arrows indicate locations of fibers in the images.

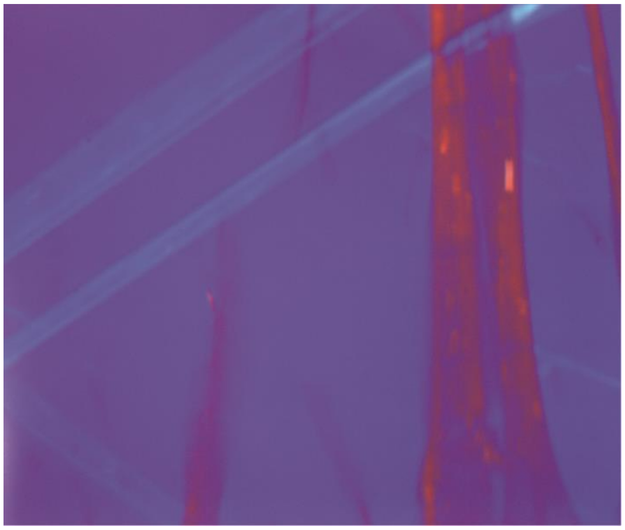

Supplementary Figure 4. Representative image of collagen fiber birefringence. Fibers with different orientations display differential refractive characteristics when subjected to polarized illumination.

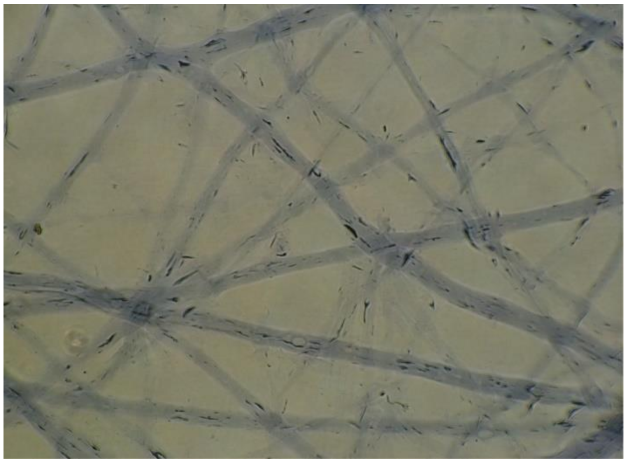

Supplementary Figure 5. Aniline blue staining of a hydrated collagen network. Staining for Aniline blue, the component of the Masson's trichrome stain responsible for staining collagen, demonstrates that the fibers formed following dextran dissolution are highly enriched in collagen.

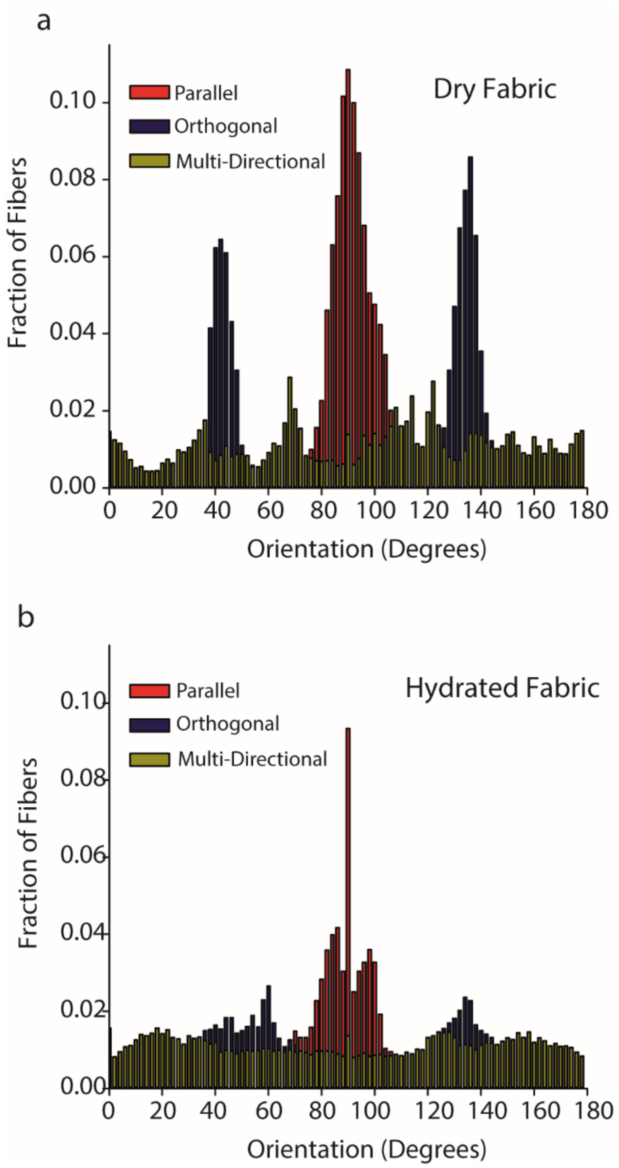

Supplementary Figure 6. Analysis of fiber orientation before and after cell culture. (a) Histogram of fiber orientation for parallel, orthogonal, and multi-direction fibers before hydration. **(b)** Histogram of fiber orientation after hydration and cell culture quantified from C1A1 images.

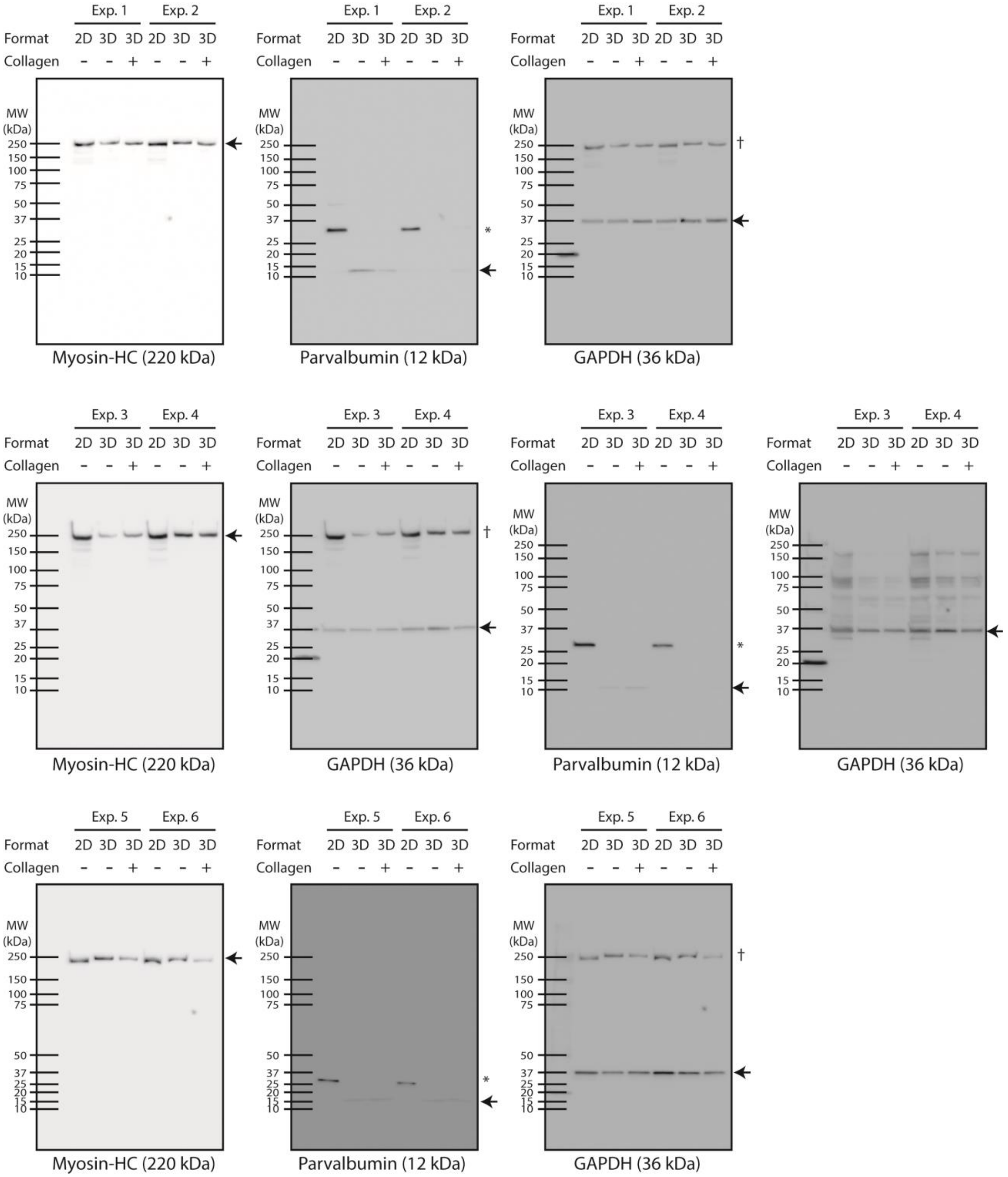
Exp. 1
Exp. 2
Format 2D 3D 3D 2D 3D 3D
Collagen - - + - - +
MW (kDa)
250
150
100
75
50
37
25
20
15
10
Myosin-HC (220 kDa)
Parvalbumin (12 kDa)
GAPDH (36 kDa)
*
†
Exp. 3
Exp. 4
Exp. 5
Exp. 6

Supplementary Figure 7. Raw Western blot data for the 6 experiments conducted to measure levels of differentiation markers under 2D and 3D growth conditions. Arrows indicate locations of bands of interest. Locations of non-specific bands for parvalbumin under 2D conditions are indicated by *. Bands present from residual myosin heavy chain on reprobed blots are indicated by †. For experiments 4 and 5, myosin heavy chain and parvalbumin were blotted from separate gels (corresponding reprobed GAPDH blots are provided). All other experiments were probed, stripped and reprobed in the following order: myosin heavy chain, parvalbumin and GAPDH. Links to relevant product data from Novus Biologicals are provided below.

Myosin heavy chain antibody: https://www.novusbio.com/products/myosin-heavy-chain-antibody-mf20_mab4470

Parvalbumin antibody: https://www.novusbio.com/PDFs/NB120-11427.pdf

GAPDH antibody: https://www.novusbio.com/products/gapdh-antibody-2d4a7_nb300-328

Supplementary Table 1. Densitometry data for myosin heavy chain and parvalbumin.

Myosin-HC (MHC)

	**MHC Exp. 1**	**GAPDH Exp. 1**	**MHC/GAPDH Exp. 1**	**MHC Exp. 2**	**GAPDH Exp. 2**	**MHC/GAPDH Exp. 2**
**2D**	5563.861	3600.619	1.545251247	6383.569	4124.083	1.547875976
**3D (-)**	2857.841	3636.205	0.785940562	4290.376	6500.619	0.659994994
**3D (+)**	3600.134	4944.79	0.728066106	3431.669	6342.205	0.541084528

	**MHC Exp. 3**	**GAPDH Exp. 3**	**MHC/GAPDH Exp. 3**	**MHC Exp. 4**	**GAPDH Exp. 4**	**MHC/GAPDH Exp. 4**
**2D**	8762.439	3579.861	2.447703696	10205.217	5197.104	1.963635325
**3D (-)**	1695.134	2799.79	0.605450409	7425.296	6655.518	1.115660118
**3D (+)**	3598.74	3200.79	1.124328681	5770.104	3665.447	1.57418836

	**MHC Exp. 5**	**GAPDH Exp. 5**	**MHC/GAPDH Exp. 5**	**MHC Exp. 6**	**GAPDH Exp. 6**	**MHC/GAPDH Exp. 6**
**2D**	5575.347	3163.719	1.762276296	9018.539	5317.326	1.696066594
**3D (-)**	8544.054	2401.184	3.558267088	7431.104	3488.962	2.129889635
**3D (+)**	4828.033	3504.548	1.377647845	3698.376	3626.619	1.019786198

Parvalbumin

	**Parv. Exp. 1**	**GAPDH Exp. 1**	**Parv./GAPDH Exp. 1**	**Parv. Exp. 2**	**GAPDH Exp. 2**	**Parv./GAPDH Exp. 2**
**2D**	313.506	3600.619	0.087070029	126.021	4124.083	0.030557338
**3D (-)**	5123.79	3636.205	1.409103722	31.95	6500.619	0.004914917
**3D (+)**	1299.062	4944.79	0.26271328	436.042	6342.205	0.068752429

	**Parv. Exp. 3**	**GAPDH Exp. 3**	**Parv./GAPDH Exp. 3**	**Parv. Exp. 4**	**GAPDH Exp. 4**	**Parv./GAPDH Exp. 4**
**2D**	110.021	5106.861	0.021543762	76.364	5389.497	0.01416904
**3D (-)**	2842.719	4394.397	0.646896264	273.092	6156.983	0.044354841
**3D (+)**	4831.083	3864.912	1.249985252	738.698	4225.154	0.174833391

	**Parv. Exp. 5**	**GAPDH Exp. 5**	**Parv./GAPDH Exp. 5**	**Parv. Exp. 6**	**GAPDH Exp. 6**	**Parv./GAPDH Exp. 6**
**2D**	65.364	3163.719	0.020660495	77.778	5317.326	0.014627277
**3D (-)**	3731.083	2401.184	1.55385135	4545.154	3488.962	1.302723847
**3D (+)**	5332.619	3504.548	1.521628181	3152.447	3626.619	0.869252326
